# The Intestinal Toxemias of Children

**Published:** 1906-08

**Authors:** L. B. Clark

**Affiliations:** Atlanta, Ga.; Lecturer on Diseases of Children, Atlanta School of Medicine


ATLANTA

### Journal-Record of Medicine

Successor to Atlanta Medical and Surgical Journal, Established 1855, and Southern Medical Record, Established 1870.

OWNED BY THE ATLANTA MEDICAL JOURNAL CO.

**Published Monthly.**

*Official organ Fulton County Medical Society, State Examining Board, Presbyterian Hospital, etc., etc.*

VOL. VIII. AUGUST, 1906. No. 5.

BERNARD WOLFF, M.D., EDITOR, Nos. 319-20 Prudential.

M. B. HUTCHINS, M.D., BUSINESS MANAGER, 1007-1008 Century Bldg.

E. G. BALLENGER, M.D., ASSOCIATE EDITOR AND ASSISTANT MANAGER.

### ORIGINAL COMMUNICATIONS.

### THE INTESTINAL TOXEMIAS OF CHILDREN.

BY L. B. CLARKE, M.D., ATLANTA, GA.

Lecturer on Diseases of Children, Atlanta School of Medicine.

I am led to present this paper through several years of observation of a class of cases, both in my own practice and in that of others, presenting an array of symptoms and manifestations peculiarly stimulating many other morbid conditions, and yet, otherwise unaccounted for under any known or hitherto recognized nomenclature.

These toxemias have not yet been properly classified in the text-books. They are made to occupy rather an insignificant position at best, when at all alluded to, and even in that king of books on medical subjects, "Holt—Infancy and Childhood," this large and exceedingly important subject is covered by only a very few remarks under the article on Gastro-enteric Intoxication, where it should not be placed. It is not an infection in the sense that gastro-enteritis is an infection.

The subject belongs, and should be described, in a class by itself. It certainly, in the cases with which I have come in contact, bears no relation to, and is not akin to summer diarrhea, and one may find nothing in common in either the symptoms or possible results. For my own part, I do not believe that the same pathology applies for the reason that I doubt the existence of morbid change in this process.

These conditions appear to have a counterpart in adult life in the form of those pseudo-typhoid conditions that have been termed toxenteric fever, or as the writer best likes—non-specific enteric fever; conditions often simulating, and mistaken for true typhoid fever.

It is true that it is only within comparatively few years that teachers like Holt have begun to properly classify these diseases; for instance, we cease to call everything relating to summer complaint cholera infantum, and we have studied out and separated the various forms of ileocolitis. It is, therefore, to be hoped that we are well on the highroad towards a completion of this work, and that more thorough nomenclature and classification await us in the near future. The author would describe the conditions under consideration as follows:

*Definition.*—An acute intestinal infection, usually accompanied by marked constipation, characterized by a tendency to hyperpyrexia, with or without prostration, no apparent lesion, and due to the absorption of toxines, either pre-existing in food or developing after ingestion.

*Etiology.*—Affects children from nine months to five years or more.

The primal factors, from the standpoint of bacterial causation, I have had no opportunity to study. From the varied clinical manifestations of the condition, undoubtedly I claim the existence of more than one form of toxemia, hence more than one cause; for the reason that, as will be seen under the head devoted to diagnosis, they resemble in one case grippe, another typhoid fever, another malaria, etc. Whatever the cause, is introduced into the alimentary canal either in the food, or toxines are developed later from the presence of some undigested, fermenting, decaying mass in the small intestine, from which absorption takes place.

*Pathology.*—In the cases in which the results of treatment verified the diagnosis, there were no fatalities, and in life no changes in any organ could be detected. In one case a fairly marked leukocytosis was found, searching for other trouble. Apparently no lesion exists, except such changes in the blood as may occur from a few days of high temperature.

*Symptoms.*—These are irregular and uncertain. The most marked symptoms presented are more or less continued high temperature and constipation. In some cases the attack is ushered in by vomiting, in others none occurs.

The physician is usually called to a child who has fever, temperature any point between 102-105. This temperature may prove more or less continuous, or in other instances may gradually fall to 99 about midnight, remaining thereabout for six to eight hours and gradually rising during morning and afternoon until it reaches 105 again, to later at night, repeat the performance, which may recur for several days, lasting from two to three to ten days, until influenced by treatment. In other attacks the temperature may reach 103 to 105 in the early morning hours and remain high during the entire day. It may be of either remittent or intermittent type, and when it intermits the paroxysm of temperature is in existence *longer* than in any known disease of that type, possibly excepting some cases of pyemia.

When the temperature is off or low the child is bright, playful; when on it is dull, stupid, sleepy, etc., yet not in all cases.

This temperature may be of a very puzzling character for several days. It may simulate typhoid fever in a somewhat irregular, step-ladder rise, only to lose that characteristic later. Careful search, however, will fail to develop any symptom of typhoid fever even up to those days when diagnosable, suspicious as it may be.

Again, a remittent, or more so, intermittent type of temperature often presented, may lead to suspect malarial fever, especially when the apparent periodicity is taken into account. The periodicity is not stable, however, and the physician soon sees that it is so irregular that it would require a dozen sets of sporulating organisms to account for it, beside not yielding to quinine.

So it is with grippe and pus. The condition may resemble either. Careful examination and re-examination will fail to disclose any cause for the temperature made more obscure if the bowels are reasonably moving.

The author recalls one case in a confrere's practice in which the symptoms ran such a course as to produce a suspicion of leukemia, until it cleared.

Constipation in most cases is marked. In others—not alone the mother, but the physician, will deny its existence. In those cases in which there is profound and obstinate constipation, it will be found that there exists an intestinal paralysis almost complete. Ordinary children's cathartics produce as much effect as so much water. It will be found necessary to use heroic dosage, almost adult doses, of calomel, castor oil, salts, etc., to produce evacuation, although high irrigation has already adduced the knowledge that there is nothing in the colon, the small intestine being the seat of trouble.

In the other type of cases, those in which there is no apparent constipation, the ordinary child doses produce stools; several daily, yet no reduction of temperature, and it is only when purging is carried to the extreme point that the desired result is obtained.

The abdomen in some cases is flat; presents no guide. In others that I have seen it is distended to its utmost, due to the paretic condition mentioned. The skin is dry, hot, tongue coated, foul in some cases, dry and leathery in others, in the majority of no value in diagnosis.

The patient may present nervous manifestations during the height of temperature. Restless tossing, delirium, all of which generally cease when the thermometer registers a falling record.

In somewhat older children skin eruptions are noted; urticaria, varied in location, intensity and duration. Kidneys often act sluggishly, and urine concentrated, such as is common in a high continued temperature. There are no other symptoms worthy of note, the condition continuing unabated until the material is removed by continuous purging, when the temperature may end by lysis or more rapidly, yet not by crisis. The physician is usually rewarded by having the child pass from the bowel some article of food undigested, the eating of which the mother denies all knowledge of. The temperature falls to normal very shortly afterward.

*Diagnosis.*—This is the most interesting feature of the condition. It is to be made from the otherwise unaccountable high temperature accompanied by more or less obstinate constipation.

It is to be differentiated from typhoid fever, from the malarial fevers, from gastro-enteritis, from pus, and the winter cases from that blanket over a multitude of sins—grippe.

*From typhoid fever* by the absence of a continual typical temperature; by the fall to normal in the intermittent type of cases—the flat abdomen existing in many cases; the absence of the typical tongue—and later the absence of spleen, lenticular rash, Widal reaction, and its early ending.

*From the malarial fevers* by the absence of the organism in the blood—the irregularity of the paroxysm—the longer duration of the paroxysm—the absence of periodicity—the therapeutic test.

*From gastro-enteritis,* differentiation should not be necessary, inasmuch as the attack present none of the symptoms of a summer diarrhea, and yet it has been so classified.

*From grippe,* with which the winter cases are most confounded (everything in winter has been denominated grippe), and otherwise good medical men fall into this error, a marked difference exists in that permanent chain inseparable from grippe. The intense prostration and cardiac weakness, disproportionate to the duration of the attack and nature of the seizure.

*From pyemia* — No marked leukocytosis, no trauma, no evidences of abscess, no involvements, age of patient, etc. This may be very difficult for several days.

Cases presenting urticaria have been diagnosed German measles. This should not be, because of the lower range of temperature in German measles, and the throat and catarrhal symptoms. Again, in one instance, the advent of the rash was after the fall of temperature to normal, which could not occur in any form of measles.

*Treatment.*—Purgation. Satisfied with the diagnosis, cleanse and recleanse the alimentary canal until the temperature is affected. Calomel, salts, castor oil, and the best of these is calomel —in successive larger doses, if need be, until desired effect. Heroic dosage may be necessary. Conjointly the colon should be irrigated with warm salt water twice or more daily. In gaseous distention turpentine irrigations will assist, with turpentine steepes on the abdomen and abdominal massage.

For the temperature, lukewarm, cool or cold sponging, as may be the necessity.

Other incidental conditions should be treated symptomatically.

The diet should be liquid—*no milk in any form.* Broths, soups (strained), albumens, peptonoid preparations, beef jelly, juices of rare steak, etc.

Great care should be exhibited in the return to food.

